# Validation of the Death and Dying Distress Scale (DADDS-Sp) in a population with advanced cancer in Chile

**DOI:** 10.3332/ecancer.2021.1326

**Published:** 2021-12-02

**Authors:** Loreto Fernández-González, Moisés Russo Namías, Rodrigo Lagos, Paulina Bravo, Alexis Troncoso, Claudia Acevedo Echeverria

**Affiliations:** 1 Instituto Oncológico, Fundación Arturo López Pérez, José Manuel Infante 805, Providencia, Región Metropolitana, 750000 Santiago, Chile; 2 Dalla Lana School of Public Health, University of Toronto, 155 College St, Toronto, ON M5T 3M7, Canada; 3 Global Institute of Psychosocial, Palliative and End of Life Care, 700 Bay Street, Suite 2303, Toronto, ON M5G 1Z6, Canada; 4 Facultad de Medicina, Universidad Diego Portales, Av Manuel Rodríguez Sur 415, 8370179 Santiago, Chile; 5 Departamento de Salud de la Mujer, Escuela de Enfermería [Department of Women’s Health, School of Nursing], Pontificia Universidad Católica de Chile, Av Libertador Bernardo O’Higgins 340, Región Metropolitana, 7820436 Santiago, Chile; 6 School of Social Sciences, Cardiff University, Glamorgan Building, King Edward VII Ave, Cardiff CF10 3NN, UK; 7 Associate Researcher, Centro Núcleo Milenio Autoridad y Asimetrías de Poder, Av Libertador Bernardo O’Higgins 3363, 71783-5 Santiago, Chile; 8 Department of Medical Technology, Faculty of Medicine, University of Chile, Avenida Independencia 1027, Independencia, Región Metropolitana, 8380453 Santiago, Chile; 9 Instituto Nacional del Cáncer, Avenida Profesor Zañartu 1010, Independencia, 13108 Santiago, Chile; a https://orcid.org/0000-0001-5026-6438; b https://orcid.org/0000-0003-0944-5244; c https://orcid.org/0000-0002-5806-6227; d https://orcid.org/0000-0001-7378-6487; e https://orcid.org/0000-0003-1970-3788; f https://orcid.org/0000-0001-7363-1362

**Keywords:** palliative care, anxiety, surveys and questionnaires, Chile

## Abstract

**Introduction:**

Developing instruments to screen for relevant aspects of advanced illness is key to identifying palliative needs and evaluating the effectiveness of interventions in this population. The objective of this project is to validate the Death and Dying Distress Scale in Spanish (DADDS-Sp) for screening anxiety about death and evaluating psychometric properties for people with advanced cancer.

**Methods:**

DADDS is a 15-item self-administered questionnaire that assesses thoughts and feelings related to death and the process of dying. A cross-sectional, descriptive, psychometric validation study was conducted in two cancer centres in Santiago de Chile. Included were patients over 18 years of age with incurable and/or metastatic cancer, fluent in Spanish, and a life expectancy of more than 3 months. Reliability was analysed using Cronbach’s alpha, and confirmatory factor analysis was performed following the model of the original scale.

**Results:**

Seventy four patients participated in the study. The median age was 63 years. Of the sample, 59% identified themselves as women. On average, participants reported low anxiety about death (mean = 21, SD = 18). Women have more death anxiety. The reliability analysis yielded a value of α = 0.93 (IC = 0.91–0.95). Factor analysis with a one-factor structure yielded Comparative Fit Index (CFI) = 0. 0.972, Root Mean Square Error of Approximation (RMSEA) = 0.092, Standardized Root Mean Square Residual (SRMR) = 0.085 and Tucker-Lewis Index (TLI) = 0.968. The model with a two-factor structure yielded CFI = 0.989, RMSEA = 0.059, SRMR = 0.075 and TLI = 0.987, suggesting that the two-factor model has a better fit for the data studied.

**Conclusions:**

DADDS-Sp is psychometrically valid for use in a Spanish-speaking population, yielding high reliability and internal consistency. A majority of the Chilean patients reported a low level of anxiety about death although about 10% presented with severe anxiety, so their identification for adequate clinical management is fundamental.

## Introduction

Optimal management of people with advanced cancer requires comprehensive management of the physical, psychosocial, spiritual and practical needs of patients and their loved ones through palliative care [1]. However, as the development of oncology has focused on remission and extending survival, palliative care has historically had difficulties positioning itself on the global agenda. Barriers to its advancement include the anthropological conceptualisation of death and human suffering [2], the prioritisation of resources on curing the disease, and the difficulty of quantifying and generating standardised indicators of its implementation, which cannot be translated into survival [3]. Therefore, the development of indicators and instruments for screening and measuring relevant aspects specific to advanced illness is key to identifying palliative needs and evaluating the effectiveness of interventions in this population.

People diagnosed with an illness with a life-limiting prognosis are confronted with the physical burden of the illness and the existential questions about their own mortality, which may trigger intense and distressing emotional responses. These reactions, added to the physical symptoms and the deterioration from advanced illness, can lead to a state of significant suffering, loss of meaning in life and a desire to hasten death [4]. To prevent and alleviate suffering in a timely manner, psychosocial assessment of the patient and their support network should be part of the standard of care for palliative care, and as such, implemented regularly and as early as possible, as soon as the patient is formally admitted to a palliative care unit or their health condition warrants an evaluation of this type [5–7].

In this context, exploring fears and thoughts about death and dying must be a central element in the assessment of a patient with palliative needs [8]. Interestingly, death anxiety has rarely been used as an outcome in studies evaluating the effectiveness of palliative care interventions. Moreover, few instruments have been specifically developed and validated in populations with advanced illness and life-limiting prognosis [9]. The proportion of patients with advanced cancer with anxiety about their own deaths can be as high as 80% [10] and may be more common than depression in this patient population. Anxiety about one’s own death is a multidimensional phenomenon, which includes fear and fantasies about the dying process and worries related to death, like the loss of time, loss of opportunities and the impact of one’s death on loved ones [11]. In order to assess death anxiety in this population, the Death and Dying Distress Scale (DADDS) was developed in Canada [9]. This questionnaire identifies the level of distress produced by the worries and challenges that emerge in the final stage of life, which can affect the ability to live life meaningfully and about which appropriate support can be offered for people with palliative needs.

Having local evidence about the psychosocial state of cancer patients with advanced illness is key for evaluating evidence-based interventions. To achieve this, valid tools are needed, that can be easily applied, and which report clinically useful data. In Chile, there are no validated instruments that allow for measuring these dimensions of the end of life in a rigorous manner. The objective of this study is to validate the DADDS Spanish (DADDS-Sp) scale and evaluate its psychometric properties for people with advanced cancer.

## Methods

Psychometric validation study, descriptive, cross-sectional, performed in two cancer centres in Santiago de Chile, one public and one private. The study was approved by a research ethics board in compliance with current regulations. Patients eligible to participate in this study were those diagnosed with advanced cancer (incurable and/or metastatic), receiving treatment with palliative intent. Inclusion criteria were being 18 years of age or older, fluent in Spanish and with a prognosis of more than 3 months. Excluded were patients with cognitive impairment or impaired consciousness, uncontrolled pain, who were hospitalised or unaware of their prognosis. Patients were invited to participate by a healthcare provider who, after explaining the objectives of the study, contacted the patient with a member of the research team. All participants signed an informed consent and individually completed the DADDS-Sp.

### DADDS-Sp

The DADDS is a self-administered questionnaire of 15 items asking about thoughts and feelings related to life, death, and dying present during the last 2 weeks [9]. The scale is divided into two sections that represent two dimensions. The first, called ‘Finitude’ includes ten questions about the distress being experienced. The second dimension (‘Dying’) includes five questions about the distress experienced about one’s own death and the process of dying. Each item must be scored from 0 to 5 on a scale of severity (0 = no distress; 1 = very little distress; 2 = mild distress; 3 = moderate distress; 4 = severe distress; 5 = extreme distress). The total score of the scale ranges from 0 to 75 points. Scores can range as mild (0 to 25 points), moderate (26 to 50 points) or severe (51 to 75 points). The DADDS has been validated and analysed for its psychometric properties and has been translated into several languages from the original English [12].

### Phases of validation

Following established methodologies [13, 14], the DADDS was first linguistically and culturally adapted from the original version in English, translated and back-translated into Spanish by translators and specialists in the subject, and parallel versions were generated that were then compared until reaching consensus. This version was validated linguistically via a pilot with three health professionals, who suggested changes to improve its legibility and comprehensibility. This version of the DADDS-Sp was piloted with ten patients (same inclusion criteria mentoned above), who later participated in cognitive interviews to evaluate its acceptability, cultural relevance and comprehensibility of the items [15]. In this way, the DADDS-Sp content was validated to proceed to psychometric validation.

### Sample size and statistical analysis plan

According to recommendations in the literature, a sample size of at least 75 participants (5 participants per item), was calculated to give an adequate analysis [16, 17]. The sample and its levels of death anxiety was characterised through descriptive statistics, presenting the averages of total scores and item averages to allow comparison between factors. To analyse the differences between groups, Chi-squared or Fisher’s Exact were used as appropriate. For the reliability analysis, the internal consistency of the items was evaluated using Cronbach’s alpha, considering an alpha equal to or greater than 0.70 as a high internal consistency. This was carried out via the alpha function of the psych package of the statistical software R, version 4.0.5 [18]. Confirmatory factor analysis (CFA) was performed following the original validation, in which two models were evaluated, a global model (all items) and a two-factor model considering the Finitude and Dying dimensions [9]. These analyses were performed with the lavaan package of the R statistical software, using pairwise maximum likelihood (PML) as the estimation method [19]. PML estimation consists of maximising marginal likelihood functions, instead of the model’s likelihood function directly, obtaining the same desired properties of a maximum likelihood estimator but at a lower computational cost and with relatively smaller sample sizes [20, 21].

## Results

### Sample characteristics

Seventy-four patients participated in the study. The median age was 63 years. 59% of the sample identified as women, and 41% as men. The majority (69%) identified as Catholic. Married or cohabitating patients predominate (62%). Almost two-thirds (65%) had been admitted to a palliative care unit, and 47% had received mental healthcare for their health situation. Finally, 55% of the sample had been diagnosed 2 years ago or less. Details can be seen in Table 1.

### DADDS-Sp results

**Figure 1.**
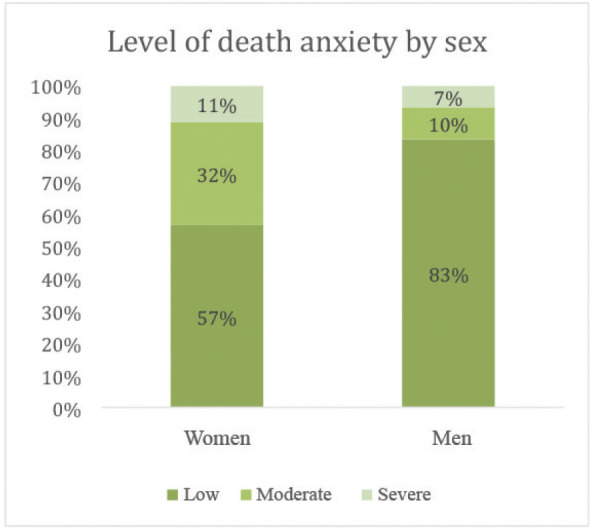
Level of death anxiety by sex.

The average of the total scores reported by the participants was 21 (SD = 18), which corresponds to a low level of death anxiety. Calculated as an average of the items, the total average is 1.4 (SD = 53) with an average of 1.3 (SD = 0.43) for the finitude dimension and 1.62 (SD = 0.65) for dying. Divided according to anxiety levels, 67.6% of the participants reported low anxiety, 22.9% rated it as moderate and 9.5% as severe. Women reported higher anxiety about death than men, in a statistically significant manner (*p* = 0.05), as shown in Figure 1. The highest scoring questions in the first section were Question 9 (‘The impact of my death on my loved ones’), followed by Question 8 (‘being a burden to others’); in the second part of the scale, Question 14 (‘[that your own death and dying may] happen with a lot of pain and suffering’) was the highest scoring question, followed by Question 12 (‘[that your own death and dying may] be prolonged or drawn out’).

### Reliability analysis of the dimensions of the DADDS-Sp scale

For the full scale, a value of *α* = 0.93 with a confidence interval of 0.91–0.95 was obtained. In addition, the effect of the loss of the items was calculated. For the Finitude dimension, *α* = 0.90 with a confidence interval of 0.87–0.93 was obtained. It should be noted that the value of ‘*α*’ remains the same or decreases if any of the items are removed, with the exception of item 9, the removal of which results in a slight increase. For the Finitude dimension, *α* = 0.97 with a confidence interval of 0.83–0.91 was obtained. In addition, the value of ‘*α*’ remains the same or decreases if any of the five items is removed (Table 2).

### CFA and internal consistency

Factor analysis with a one-factor structure yielded Comparative Fit Index (CFI) = 0.972, Root Mean Square Error of Approximation (RMSEA) = 0.092, Standardized Root Mean Square Residual (SRMR) = 0.085 and Tucker-Lewis Index (TLI) = 0.968. The model with a two-factor structure yielded CFI = 0.989, RMSEA = 0.059, SRMR = 0.075 and TLI = 0.987, suggesting that the two-factor model has a better fit for the data studied.

On the other hand, when comparing both models by their Akaike information criterion (AIC) (one factor = 59,378.86; two factors = 59,408.02), no statistically significant difference was observed (*p*-value = 0.60) (Table 3).

Table 4 shows the standardised weight for each factor. It is important to note that all of these values are statistically significant (*p*-value < 0.05).

## Discussion

This study presents the first valid instrument to measure death anxiety in the Chilean population with advanced cancer, the DADDS, version DADDS-Sp. Screening for death anxiety as part of the clinical assessment of the palliative patient will facilitate identifying and addressing this phenomenon.

The results presented are consistent with the validation of the instrument in its original language, in which it is possible distinguish two factors associated with the expression of anxiety about death [9].

Due to the sample size, the PML estimation method was chosen instead of maximum likelihood (ML). PML presents properties similar to ML and requires a smaller sample size. A comparison between the one-factor and two-factor models shows that the two-factor model has a better fit. However, when comparing AICs they are not statistically significant. This is possibly due to the statistical power given by a relatively small sample size, but it is undeniable that the two-factor model is more appropriate, also considering the reliability analysis and the psychometric properties of the original scale.

In the present work with Chilean patients, CFA identifies the same two factors as the original psychometric model, Finitude and Dying. However, descriptively the results are different: specifically, in the Canadian study, a higher level of anxiety is observed than in the Chilean sample. This may be because the participants in that study were recruited as a part of a clinical trial of psychotherapy in patients with advanced cancer, so they may have been emotionally distressed and in search of interventions for relief and management. In the validation and adaptation for the German population, it was decided to eliminate several items related to the Dying factor, in order to increase its acceptability, which transforms it into a one-factor scale [12]. In the Chilean version, the Dying factor has a higher mean score than the Finitude factor, which shows that it is a clinically relevant aspect and that, although it may be difficult to answer for some people, it is important to screen it and make it visible. Moreover, both the DADDS and the DADDS-Sp are able to discriminate between mild, moderate and severe death anxiety, showing that its use allows the identification of cases with clinically significant distress [22].

Discussion of death anxiety with healthcare providers can facilitate advanced planning, avoiding actions such as invasive procedures and hospitalisations via emergency services at the end of life, favouring the use of hospice or home care [23]. In spite of historical misgivings about the possible iatrogenic effects of talking about advanced planning with these patients, research in this area has made it possible in recent years to refute this hypothesis [24], and has demonstrated the acceptability of interventions that involve their identification and management [25].

A concrete tool, like a questionnaire, can be a non-threatening start to explore topics that can be tremendously sensitive for individuals who find themselves in situations of health-related suffering. Similarly, it may help clinicians and other healthcare providers to talk about situations that may be difficult to name, or sometimes even to imagine. Furthermore, the use of tools in healthcare helps systematise clinical practice between services, as much within one institution as between institutions. Palliative care, as a service provided throughout the country at different levels of care, can be highly variable. The patients’ needs, however, are often universal. The need to be treated with dignity, and in accordance with one’s own personal choices and values, is a human right whose variability in the quality of care should be minimised as much as possible [1].

In the current national debate about access to palliative care and euthanasia, it is critical to have local evidence about the psychological suffering associated with dying for people in advanced stages of cancer illnesses. Only in this way will it be possible to offer services that are appropriate to the needs of the population, in a health context that foresees an exponential increase in the number of people in need of palliative care in the country.

The present study has strengths and limitations. Among its strengths – it constitutes a relevant and novel project, providing a psychometrically valid version of the DADDS for the Spanish-speaking population. In addition, the sample includes participants with varied clinical and sociodemographic profiles from both public and private health systems. As far as weaknesses, on the other hand, the participants were recruited from two cancer centres in Santiago de Chile, so their answers may not be representative of other populations who receive care in other cities and centres in the country. On the other hand, the sample is relatively small, consisting of the minimum sample size originally planned, although the analyses take this aspect into account. However, the recruitment was conducted entirely prior to the COVID-19 pandemic, and it was the team’s decision to end the data collection at the beginning of the pandemic given the health risks and the possible bias of including patients whose mental health was affected by the effects of the pandemic, prioritising the homogeneity of the sample.

## Conclusions

The Spanish version of the DADDS scale, DADDS-Sp, is psychometrically valid for use in a Spanish-speaking population. DADDS-Sp possesses high reliability and internal consistency, and maintains the two-factor model of the original scale. Screening for anxiety about death and dying can be performed on patients with advanced cancer through this 15-item self-administered questionnaire. While most Chilean patients reported a low level of anxiety about death, about 10% reported severe anxiety, and identification for clinical management is fundamental to alleviate this distress.

## Conflicts of interest

The authors of the manuscript have no conflicts of interest.

## Source of funding

Financial support for the writing of this article was received from the National Fund for Research and Development in Health (FONIS, according to its Spanish acronym) N° SA18I0058. FONIS had no influence on the design, collection or analysis of the data, nor in the preparation or approval of the current manuscript.

## Figures and Tables

**Table 1.** Description of the sample.

Gender		
	Male	30 (41%)
	Female	44 (59%)
Age		63 (28–91)
Institution		
	Private	48 (65%)
	Public	26 (35%)
Religion		
	Catholic	51 (69%)
	Other Christian	13 (18%)
	Other	6 (8%)
	Agnostic/None	4 (5%)
Marital status		
	Married/cohabitating	46 (62%)
	Separated	5 (7%)
	Single	17 (23%)
	Widowed	6 (8%)
Number of persons with whom you live		
	None	2 (3%)
	1–3	50 (67%)
	4 or more	22 (30%)
Identify caregiver		
	Yes	37 (64%)
	No	27 (36%)
Education		
	School incomplete	20 (27%)
	School complete	16 (22%)
	Technical/university	28 (51%)
Insurance		
	Private	16 (22%)
	Public	58 (78%)
Primary tumour site		
	Breast	15 (20%)
	Skin	3 (4%)
	Lung	12 (16%)
	Intestinal	15 (20%)
	Genitourinary	16 (22%)
	Gynaecological	8 (11%)
	Other	5 (7%)
ECOG		
	0	25 (34%)
	1	37 (50%)
	2	10 (14%)
	3 or 4	2 (2%)
Admitted to palliative care		
	No	26 (35%)
	Yes	48 (65%)
Mental health history		
	No	39 (53%)
	Yes	35 (47%)
Current treatment		
	Systemic	29 (40%)
	Radiotherapy	29 (40%)
	Both	13 (18%)
Time since diagnosis		
	<2 years	41 (55%)
	>2 years	33 (45%)

**Table 2.** Reliability analysis.

		Total scale	Factor 1: Finitude		Factor 2: Death
of Cronbach		0.926	0.899	0.868	
**of Cronbach per item deleted**					
**Code**	**Item**				
dadds1	Not having done all the things I wanted to do	0.921	0.883	-	
dadds2	Not having said all that I wanted to say to the people I care about	0.926	0.899	-	
dadds3	Not having achieved my life goals and ambitions	0.925	0.895	-	
dadds4	Not knowing what happens near the end of life	0.920	0.883	-	
dadds5	Not having a future	0.919	0.879	-	
dadds6	The missed opportunities in my life	0.923	0.888	-	
dadds7	Running out of time	0.918	0.880	-	
dadds8	Being a burden to others	0.923	0.891	-	
dadds9	The impact of my death on my loved ones	0.924	0.901	-	
dadds10	My own death and dying	0.919	0.888	-	
dadds11	Happen suddenly or unexpectedly	0.919	-	0.836	
dadds12	Be prolonged or drawn out	0.921	-	0.832	
dadds13	Happen when I am alone	0.923	-	0.859	
dadds14	Happen with a lot of pain or suffering	0.924	-	0.846	
dadds15	Happen very soon	0.918	-	0.824	

**Table 3.** Test for comparison of the two models.

	Df	PL_AIC	PL_BIC	Chisq	Chisq diff	Df diff	Pr (>Chisq)
Two	89	59378.86	60515.48	111.7050			
Global	90	59408.02	60538.87	145.8882	0.2653939	1	0.6064384

Chisq: Chi-Squared statistic

**Table 4.** CFA.

Code	Item	One factor	One factor: Finitude	One factor: Death
dadds1	Not having done all the things I wanted to do	1.136 (0.163)	1.171 (0.154)	-
dadds2	Not having said all that I wanted to say to the people I care about	0.807 (0.189)	0.821 (0.193)	-
dadds3	Not having achieved my life goals and ambitions	0.763 (0.196)	0.785 (0.201)	-
dadds4	Not knowing what happens near the end of life	1.209 (0.208)	1.247 (0.206)	-
dadds5	Not having a future	1.267 (0.204)	1.314 (0.2)	-
dadds6	The missed opportunities in my life	0.952 (0.224)	0.99 (0.226)	-
daddsa7	Running out of time	1.29 (0.168)	1.322 (0.16)	-
dadds8	Being a burden to others	1.088 (0.174)	1.113 (0.171)	-
daddsa9	The impact of my death on my loved ones	1.302 (0.192)	1.297 (0.189)	-
dadds10	My own death and dying	1.443 (0.189)	1.427 (0.186)	-
dadds11	Happen suddenly or unexpectedly	1.198 (0.194)	-	1.308 (0.186)
dadds12	Be prolonged or drawn out	1.225 (0.177)	-	1.348 (0.161)
dadds13	Happen when I’m alone	0.989 (0.231)	-	1.095 (0.227)
dadds14	Happen with a lot of pain or suffering	1.11 (0.193)	-	1.258 (0.177)
daddsb5	Happen very soon	1.477 (0.185)	-	1.629 (0.165)
